# Roles of c-FLIP in Apoptosis, Necroptosis, and Autophagy

**DOI:** 10.4172/2157-2518.S6-003

**Published:** 2013

**Authors:** Ahmad R Safa

**Affiliations:** 1 Department of Pharmacology & Toxicology, Indiana University School of Medicine, IN 46202, USA; 2 Indiana University Simon Cancer Center, Indiana University School of Medicine, IN 46202, USA

**Keywords:** c-FLIP, apoptosis, death receptors, cancer, chemotherapy

## Abstract

Cellular FLICE (FADD-like IL-1β-converting enzyme)-inhibitory protein (c-FLIP) is a major antiapoptotic protein and an important cytokine and chemotherapy resistance factor that suppresses cytokine- and chemotherapy-induced apoptosis. c-FLIP is expressed as long (c-FLIPL), short (c-FLIPS), and c-FLIPR splice variants in human cells. c-FLIP binds to FADD and/or caspase-8 or -10 and TRAIL receptor 5 (DR5). This interaction in turn prevents Death-Inducing Signaling Complex (DISC) formation and subsequent activation of the caspase cascade. c-FLIPL and c-FLIPS are also known to have multifunctional roles in various signaling pathways, as well as activating and/or upregulating several cytoprotective and pro-survival signaling proteins including Akt, ERK, and NF-κB. In addition to its role in apoptosis, c-FLIP is involved in programmed necroptosis (necrosis) and autophagy. Necroptosis is regulated by the Ripoptosome, which is a signaling intracellular cell death platform complex. The Ripoptosome contains receptor-interacting protein-1/Receptor-Interacting Protein-3 (RIP1), caspase-8, caspase-10, FADD, and c-FLIP isoforms involved in switching apoptotic and necroptotic cell death. c-FLIP regulates the Ripoptosome; in addition to its role in apoptosis, it is therefore also involved in necrosis. c-FLIPL attenuates autophagy by direct acting on the autophagy machinery by competing with Atg3 binding to LC3, thereby decreasing LC3 processing and inhibiting autophagosome formation. Upregulation of c-FLIP has been found in various tumor types, and its silencing has been shown to restore apoptosis triggered by cytokines and various chemotherapeutic agents. Hence, c-FLIP is an important target for cancer therapy. This review focuses on (1) the anti-apoptotic role of c-FLIP splice variants in preventing apoptosis and inducing cytokine and chemotherapy drug resistance, as well as its roles in necrosis and autophagy, and (2) modulation of c-FLIP expression as a means to enhance apoptosis and modulate necrosis and autophagy in cancer cells.

## Introduction

Various cell death signaling pathways have been described that are regulated at several levels. While the mitochondrion is the initiation point of these signals [1,2], there are several types of signaling platforms in cells that can initiate cell death. These complexes include the death-inducing signaling complex (DISC) [3], TNF complex II [4], apoptosome [5], PIDDosome [6], and Ripoptosome [7,8]. Interestingly, the c-FLIP isoforms, c-FLIPL, c-FLIPS, and c-FLIPR, regulate apoptosis [by interacting with the death signaling complex downstream of TNF-α receptors, Fas, and TRAIL receptors 1 (DR4) and 2 (DR5), necroptosis, and autophagy]. In this review, I discuss (1) apoptosis signaling pathways and the role of c-FLIP isoforms as critical anti-apoptotic and drug resistance factors, (2) the roles of c-FLIP isoforms in regulating necrosis and autophagy, and (3) the potential for improving the outcome of cancer therapy by targeting c-FLIP isoforms.

## Apoptosis Signaling Pathways

**Figure 1:**
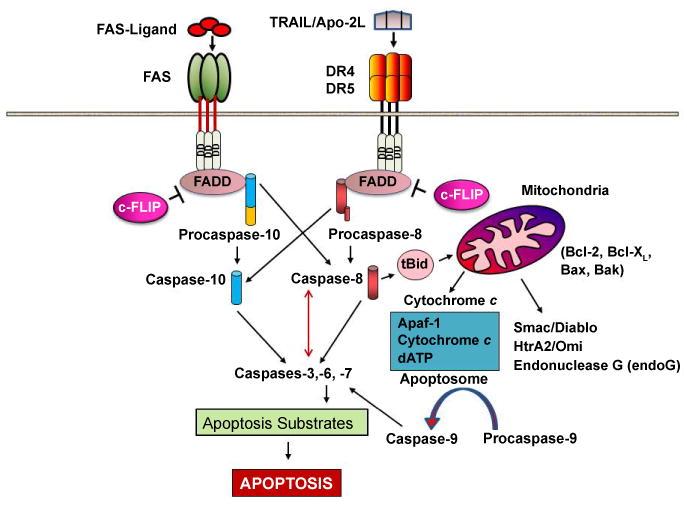
Apoptosis signaling pathways and roles of c-FLIP in preventing apoptosis. Interaction of TRAIL with its receptors DR4 and DR5 or binding of Fas ligand to Fas receptor initiates the death receptor (extrinsic) and subsequently mitochondrial apoptosis signaling pathways through FADD-dependent autocatalytic activation of caspases-8 and -10 and Bid cleavage to truncated Bid. c-FLIP isoforms suppress caspase-8 and -10 activation, therefore preventing the downstream apoptosis cascade.

Two main signaling pathways, the intrinsic or mitochondrion-initiated pathway and the extrinsic or cell surface death receptors pathway, regulate apoptosis (Figure 1) [1,2,9,10]. In the intrinsic pathway, cytochrome c, apoptosis-inducing factors including Smac/DIABLO, HtrA2/Omi, and Endonuclease G (endoG) are released from the mitochondrial to the cytosol [11,12]. Upon release, cytochrome c and dATP bind to apoptotic proteinase-activating factor-1 (Apaf-1), and this complex along with adenine nucleotides form the apoptosome and promote procaspase-9 autoactivation [12,13,14]. Apoptosome assembly is a crucially important point in the mitochondrial pathway of apoptosis, consisting of a wheel-like heptamer of seven Apaf-1 molecules and seven cytochrome c molecules that bind and activate the initiator caspase-9, which in turn activates caspases-2, -3, -6, -7, -8, and -10 [13,14]. Apoptosis induced by different death stimuli requires direct activation of Bax and BAK at the mitochondria by a member of the Bcl-2 homology domain-3 (BH3)-only family of proteins including Bid, Bim, or PUMA [15]. The various anti- and pro-apoptotic members of the Bcl-2 family form an interactive network that finally regulates the release of apoptosis triggering factors such as cytochrome c to the cytoplasm [16]. This release of cytochrome c is associated with opening the Permeability Transition Pore (PTP) and a collapse of mitochondrial transmembrane potential (Δψm) due to the intake of Ca^2+^ following its release into the cytosol from the Endoplasmic Reticulum (ER) [9, 17]. In the death receptor-mediated or extrinsic apoptosis pathway (e.g., Fas/Fas ligand interaction, tumor necrosis factor α [(TNF-α)/TNF receptor 1 (TNFR1), or TRAIL/DR5 interaction and cell death], the initiator caspases-8 and -10 activate the downstream caspases including caspases-3, -6, and -7 [10,18,19]. Active caspases-8 and -10 are known to cleave a pro-apoptotic Bcl-2 family member, Bid, and the truncated Bid induces mitochondrial cytochrome c release [19-23], thereby linking the two pathways. After activation, both caspases-8 and -9 activate caspase-3, which in turn cleaves other caspases and many cellular proteins [24-26]. Scaffidi et al. [27,28] also have identified two different CD95 apoptosis signaling cell types, type I and type II. In type I cells, CD95-mediated apoptosis is initiated by large amounts of active caspase-8 formed at the DISC and subsequent direct cleavage of caspase-3. In contrast, in type II cells, very little DISC formation and small amounts of active caspase-8 sufficient to trigger the mitochondrial apoptosis pathway lead to a significant activation of both caspase-8 and caspase-3. Overexpressed Bcl-2 or BclxL only can block apoptosis in type II cells [28]. These authors [28] have shown that several apoptosis-inhibiting or -inducing stimuli only affect apoptosis in type II cells. Interestingly, since c-FLIP acts directly at the DISC, it blocks CD95-mediated apoptosis in both type I and type II cells [28].

## Cellular FLICE-like Inhibitory Protein (c-FLIP)

**Figure 2:**
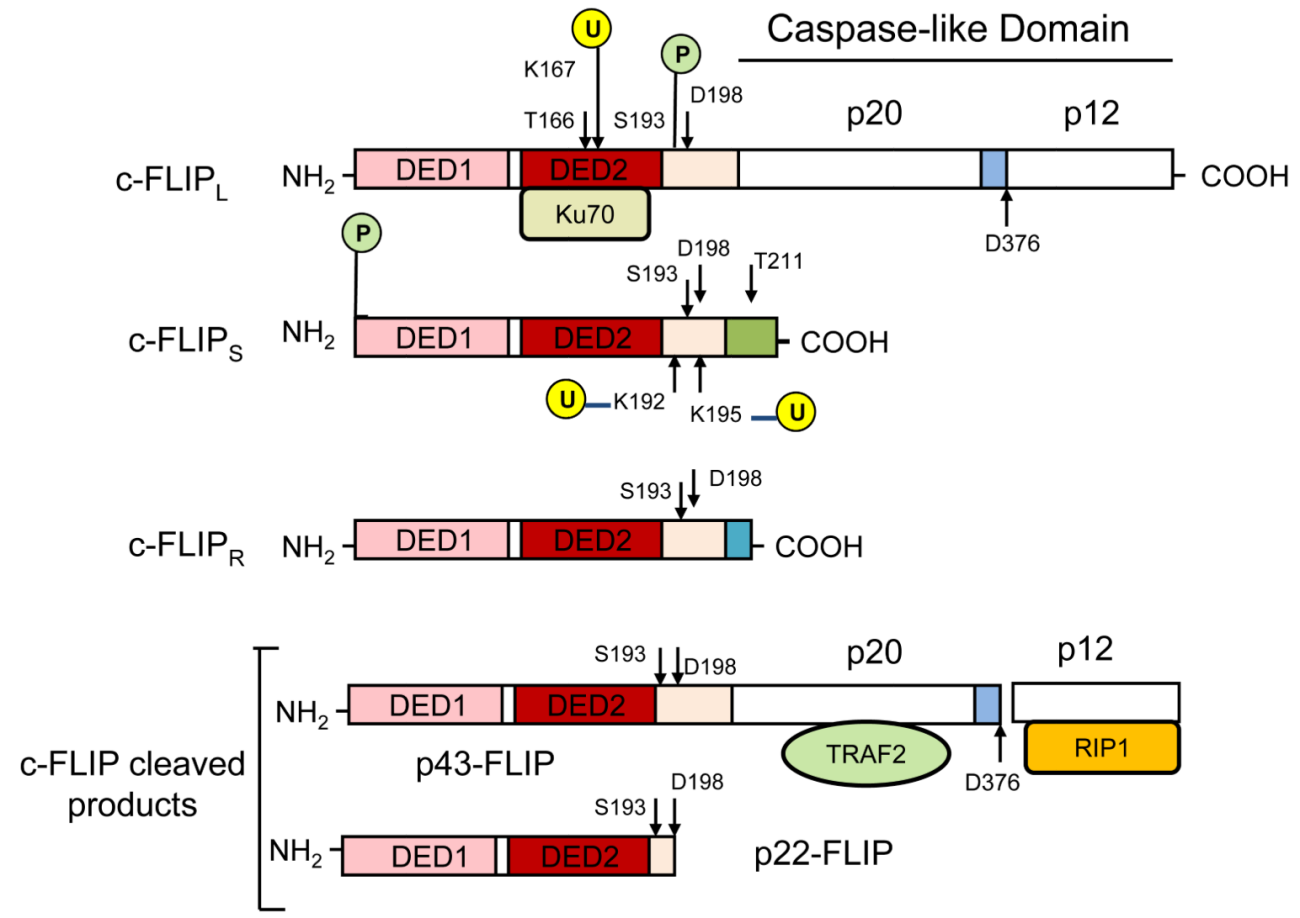
Structures of c-FLIP variants and cleavage products. c-FLIP isoforms (c-FLIPL, c-FLIPS, and c-FLIPR) contain two death effector domains (DED1 and DED2) at their N termini which are required for DISC recruitment. In addition to two DEDs, c-FLIPL has a significant similarity to caspase-8 and has a large (p20) and a small (p12) caspase-like domain which are catalytically inactive. c-FLIPS and c-FLIPR consist of two DEDs and a small C terminus. c-FLIPL can be cleaved by caspase-8 generating the N-terminal fragment p43-FLIP or p22-FLIP. The phosphorylation (P) sites and ubiquitination (U) sites are indicated [35,37,60]. The p20/p12 regions interact with TRAF2 and RIP1, respectively, and Ku70 binds to DED2 [57].

Viral FLICE-Inhibitory Proteins (v-FLIPs) were first identified by a bioinformatic search for a novel death effector domain (DED-containing virus-encoded apoptotic regulatory proteins) by Thome et al. [29]. These authors described six v-FLIPs and showed that cells expressing v-FLIPs were resistance to Fas (CD95/APO-1)-, TRAILR1-, and TNFR1-Induced Apoptosis [29,30]. v-FLIPs contain two DEDs, bind to the DED of FADD and interfere with the FADD-procaspase 8 interaction, leading to suppressed recruitment of procaspase-8 to the DISC and its activation [29]. Shortly after the characterization of v-FLIPs, the mammalian cellular homologue was identified and termed c-FLIP [31]. c-FLIP is also known as Casper, iFLICE, FLAME-1, CASH, CLARP, MRIT, or usurpin [32]. c-FLIP consists of 13 distinct spliced variants, three of which are expressed as proteins: the 26 kDa short form (c-FLIPS), the 24 kDa form of c-FLIP (c-FLIPR), and the 55 kDa long form (c-FLIPL) [33-37] (Figure 2). The three c-FLIP variants can interact with the adaptor protein FADD. The structures of c-FLIPS and the viral FLIP (v-FLIP) proteins are similar, except that the two DEDs of c-FLIPS are followed by 20 amino acids that appear to be crucial for its ubiquitination and targeting for proteasomal degradation [32,34-36]. c-FLIPR also contains two DEDs but lacks the additional carboxy (C)-terminal amino acids that are present in c-FLIPS. The C-terminus of c-FLIPL is longer than that of c-FLIPS and closely resembles the structure of caspases-8 and -10 [32,34-36], but this region of c-FLIPL does not contain a functional caspase domain. This lack of caspase activity is the result of several amino acid substitutions, particularly the crucial cysteine residue in the catalytic domain which is necessary for the catalytic activity of caspases [32,34]. Additionally, c-FLIPL has a caspase-8 cleavage site at position Asp-376 (LEVD); c-FLIPL cleavage at this site produces the proteolytic fragment variant p43c-FLIP [18,19]. The C-terminal region of c-FLIPS and c-FLIPR play a crucial role in ubiquitination and degradation as well as the anti-apoptotic function of these isoforms [18,19,36]. In humans, a single nucleotide polymorphism in a 3' splice site of the c-FLIP gene determines whether c-FLIPS or c-FLIPR is made [37]. An intact splice site directs production of c-FLIPS, but the splice-dead variant causes production of c-FLIPR [37].

## c-FLIP Function

c-FLIP isoforms are Death Effector Domain (DED)-containing proteins that are recruited to the DISC and regulate activation of caspases-8 and -10 in the death receptor signaling pathways (Figure 1). While the functional roles of c-FLIPL and c-FLIPS in apoptosis are well documented, their differential roles in cancer are not well established. Moreover, the function of c-FLIPR in cancer is poorly understood. Recent results demonstrated that expression of c-FLIPR in hematopoietic cells supports an efficient immune response against bacterial infections [37]. In humans a 3' splice site of the c-FLIP gene (rs10190751A/G) determines whether either c-FLIPS or c-FLIPR is produced [38].

## Regulation of c-FLIP Expression

It is well established that c-FLIP is regulated both at the transcriptional and posttranscriptional levels by various stimuli [34,38]. A diverse group of transcription factors are known to transcriptionally regulate the c-FLIP gene [19,34,36,39-47]. The expression of c-FLIP is regulated at the transcriptional, translational, and post-translational levels [19,34,36]. The transcriptional control of c-FLIP isoforms is quite complex and differentially regulated in various cells in a signal-specific manner. While numerous transcription factors trigger c-FLIP transcription including NF-κB, AP-1 (cFos/c-Jun), P53, FoxO, CREB, NFATc2, EGR1, AR, SP1 [19,34,36,39-43], and p63 (NP63) [40], others induce repression of c-FLIP expression such as c-Fos, c-myc, FoxO3a, IRF5, and SP3 [44-47]. E2F1 transcription factor also downregulates c-FLIPS in lung adenocarcinoma [48]. Moreover, in the presence of androgens prostate apoptosis response factor-4 (Par-4) is recruited to the c-FLIP promoter and enhances c-FLIP expression in prostate cancer cells [41]. Viral proteins are capable of targeting cellular signaling pathways that regulate cellular non-specific immune responses and cell death. In this regard, Halder et al. [49] recently demonstrated that influenza A virus matrix protein 1 (M1) activates RelB (an NF-κB member)-mediated survival genes (c-FLIP, cIAP1, and cIAP2).

## Post-Transcriptional Regulation of cFLIP Expression

c-FLIPS can also be upregulated at the translational level and cause TRAIL resistance in glioblastoma multiforme (GBM) cells due to activation of the Akt mammalian target of rapamycin (mTOR)-p70 S6 kinase 1 (S6K1) pathway [50,51]. Conversely, inhibition of mTOR or its target S6K1 suppressed polyribosomal accumulation of c-FLIPS mRNA, c-FLIPS protein expression, and promoted TRAIL resistance in GBM cells. Moreover, it has been shown that Rocaglamide (Roc) sensitizes resistant adult Tcell leukemia/lymphoma (ATL) cells to DR4- and DR5-mediated apoptosis by translational suppression of c-FLIPS through inactivation of the translation initiation factor 4E (eIF4E) [52]. c-FLIP variants are short-lived proteins that are predominately degraded by the ubiquitin-proteasome degradation system. Both c-FLIP isoforms can be degraded by the proteasome. However, c-FLIPS is particularly sensitive to ubiquitination and proteasomal degradation, partly due to two crucial lysine residues in the C-terminal 20 amino acids that are unique to c-FLIPS [53]. c-FLIPL and c-FLIPS levels are also regulated by JNK activation via the E3 ubiquitin ligase Itch [54]. Phosphorylation events also play important roles in regulating c-FLIP protein levels. For instance, protein kinase C phosphorylation at the serine 193 (S193) residue of c-FLIPS inhibits its polyubiquitination, stabilizes c-FLIPS levels, and increases cell survival [55]. S193 phosphorylation is markedly increased by treatment with the PKC activator 12-Otetradecanoylphorbol-13-acetate and decreased by inhibition of PKCα and PKCβ. Phosphorylation of the S193 residue also decreased the ubiquitination of c-FLIPL but did not affect its stability, indicating that S193 phosphorylation has a different function in c-FLIPL than in c-FLIPS. Kerr et al. [56] reported a novel interaction between c-FLIP and the DNA repair protein Ku70 and showed that Ku70 regulates FLIP protein stability by inhibiting its polyubiquitination. Moreover, these authors demonstrated that HDAC6 is a critical regulator of Ku70 acetylation and c-FLIP protein stability [57,58]. Reactive Oxygen Species (ROS) have been implicated in the degradation of c-FLIP protein [38,39]. Recently, Wilkie-Grantham et al. [59] uncovered the mechanism by which ROS post-transcriptionally regulate c-FLIP protein levels in prostate cancer cells. They also identified threonine 166 as a novel phosphorylation site and showed that Thr-166 phosphorylation is required for ROS-induced Lys-167 ubiquitination. These authors identified lysine 167 as a novel ubiquitination amino acid of c-FLIPL which is important for ROS-dependent degradation and regulates both the stability of c-FLIP and the sensitivity of cancer cells to TRAIL.

## c-FLIP inhibits apoptosis

c-FLIP is involved in TRAIL, Fas, TNF-α, and chemotherapeutic drug resistance in a wide range of human malignancies [19,32,34-36]. Moreover, many studies encompassing diverse types of human cancer cells have revealed that the role of c-FLIP in cancer cells is anti-apoptotic. Furthermore, interference with c-FLIP expression sensitizes tumor cells to death ligands and chemotherapy in experimental models [10,25]. In addition to its function as an apoptosis modulator, c-FLIP exerts other cellular functions including increased cell proliferation and tumorigenesis [19,34-36].

The structural differences between human c-FLIP variants indicate distinct regulatory roles for c-FLIPL and c-FLIPS in apoptosis. All three c-FLIP isoforms have two DED domains and can bind to the DISC. c-FLIPS and c-FLIPR block procaspase-8 activation and apoptosis [18,19,60-62]. In a similar way to c-FLIPS, c-FLIPL acts as an anti-apoptotic molecule when it is present at high concentrations at the DISC [18,63]. c-FLIPL can also act as a pro-apoptotic molecule under strong death receptor stimulation, or in the presence of high levels of c-FLIPS or c-FLIPR which promotes the activation of procaspase-8 at the DISC [11,18,63]. We have previously demonstrated that c-FLIPL or c-FLIPS prevents caspase-8 and -10 activation [64]. We also have demonstrated that c-FLIPL interacts with DR5, FADD, and caspase-8 forming an Apoptotic Inhibitory Complex (AIC) in MCF-7 breast cancer cells [45]. Moreover, silencing the c-FLIP gene by a specific siRNA leads to death ligand-independent but DR5-, FADD-, and caspase-8- and -9-dependent apoptosis in these cells, and the knockdown of c-FLIP expression inhibits breast cancer cell proliferation and triggers spontaneous apoptosis by activating both the death receptor and mitochondrial pathways [64]. Jin et al. [65] have demonstrated that the peptide corresponding to the DR5 binding domain of c-FLIPL induces apoptosis in cancer cells. It is possible that inhibiting the interaction of DR5 and c-FLIPL by peptides or small molecule inhibitors could provide a mechanism by which tumor-selective apoptosis can be achieved. The dynamic interaction between procaspase-8 and c-FLIP isoforms at the DISC and regulation of apoptosis have been thoroughly reviewed [18,32,36,66].

## Upregulation of c-FLIP in Human Cancers

c-FLIP has been found to be overexpressed in several types of malignancies and could be associated with cancer progression due to its ability to inhibit the apoptotic process. Elevated expression levels of c-FLIP have been reported in colorectal cancer [67,68], bladder urothelial cancer [69], cervical cancer [70], Burkitt's lymphoma [71], non-Hodgkin's lymphoma [72], Head and Neck Squamous Cell Carcinoma (HNSCC) [73], hepatocellular cancers [74], and high-grade prostatic intraepithelial neoplasia (HGPIN) with maximal c-FLIP expression detected in Castrate-Resistant Prostate Cancer (CRPC) [75]. c-FLIP upregulation is also seen in gastric cancer and plays an important role in lymph node metastasis, which ultimately contributes to tumor progression [76]. c-FLIP variants are also expressed in pancreatic intraepithelial neoplasms as well as pancreatic ductal adenocarcinomas, while c-FLIP was not detected in normal pancreatic ducts [77]. While the epithelium of the normal cervix and low-grade squamous intraepithelial lesions were mainly negative for c-FLIP, high-grade intraepithelial lesions as well as cancer samples showed increased c-FLIP expression [78]. This finding suggests that c-FLIP expression may serve as a potential cervical cancer progression marker. Recent results demonstrated that c-FLIP may play an important role in the potential of osteosarcoma to metastasize to the lung [79]. McLornan et al. [80] have provided evidence that c-FLIP may also serve as a prognostic biomarker in Acute Myeloid Leukaemia (AML). While overexpression of the c-FLIPS variant has been reported in human lung adenocarcinomas with low levels of E2F1, c-FLIPL protein expression was not altered [48]. Moreover, Ueffing et al. [39] demonstrated that an increased lymphoma risk is associated with the rs10190751 A genotype causing c-FLIPR expression.

## Roles of c-FLIP in Necroptosis and Autophagy

**Figure 3:**
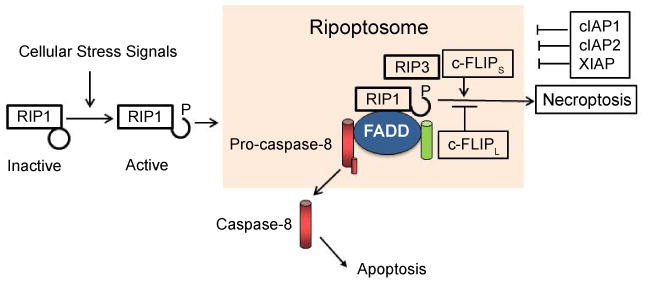
Activation and signaling by the Ripoptosome and its regulation by c-FLIP isoforms. Cellular stress and IAPs proteasomal degradation after treating cells with IAP antagonists triggers formation of the Ripoptosome, a complex of active RIP1, FADD, caspase-8/10, and c-FLIP isoforms). Its formation is independent of the mitochondrial and death receptor pathways. c-FLIPL inhibits. FLIP isoforms. Its formation is independent of the mitochondrial and death receptor pathways. C-FLIPL inhibits Ripoptosome formation [7, 8], while c-FLIPS promotes Ripoptosome assembly [8]. IAPs inhibit Ripoptosome-mediated apoptosis and necrosis [7,8].

In addition to its role in apoptosis, c-FLIPL also plays an important role in necroptotic cell death [7,8] and autophagy [81-83]. Moreover, vFLIP also plays a major role in autophagy [81,84-86]. Recent results have demonstrated that necroptosis is a programmed receptor-interacting protein 1/receptor-interacting protein-3 (RIP-1/RIP-3)-dependent necrotic cell death induced when caspase-8 activation is inhibited [88]. Previous reports have shown that c-FLIPL regulates necroptosis through the formation of the signaling platform Ripoptosome, a RIP1/caspase-8-containing intracellular cell death complex [7,8]. Moreover, the cellular inhibitor of apoptosis proteins (cIAPs) block Ripotosome formation. While c-FLIPL prevents Ripoptosome formation, interestingly, c-FLIPS promotes its assembly (Figure 3). Therefore, c-FLIP isoforms in the Ripoptosome determine whether cell death occurs by RIP3-dependent necroptosis or caspase-dependent apoptosis [8].

**Figure 4:**
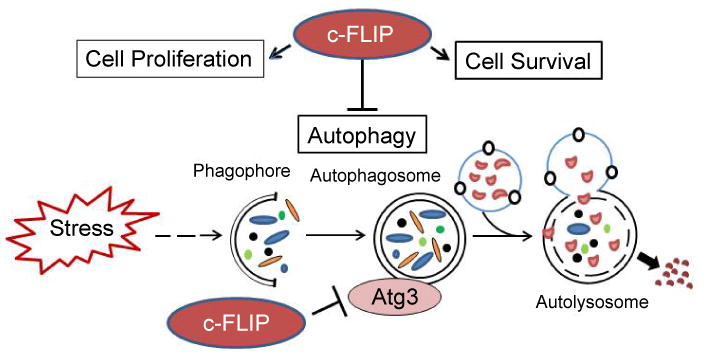
c-FLIPL mediates regulation of autophagy. Upon cellular stress, autophagosome formation can be induced. c-FLIPL attenuates autophagy by directly acting on the autophagy machinery by inhibiting Atg3 binding to LC3, thereby decreasing LC3 processing.

Autophagy is a degradation pathway by which cytoplasmic components including various damaged cytoplasmic organelles and unused long-lived proteins are sequestered in the autophagosomes, which are delivered to the lysosome to form an autolysosome for final degradation (Figure 4). Autophagy as a programmed cell survival mechanism is important in regulating and maintaining several normal human physiological processes [88-90]. Autophagy also is implicated in the pathogenesis of several diseases in human including cancer, neurodegenerative diseases, aging, muscle diseases, infection, and immunological diseases [90-96]. While autophagy can suppress tumor growth, it also enables tumor cells to survive under stress [97]. Suppression of autophagy can also sensitize cancer cells to anticancer therapy [99,100], but under the apoptosis deficiency condition, autophagy can also cause cell death termed “autophagic cell death” [95,100,94].

The autophagy pathway is controlled by complex molecular machinery [101,102]. Beclin-1, its binding partner class III phosphoinositide 3-kinase (PI3K) and other cofactors [103] form the complex termed Vps34 which is required for initiating formation of the autophagosome in autophagy [104,105]. During autophagosome formation, the microtubule-associated protein 1 Light Chain 3 (LC3) ubiquitin-like protein is cleaved by the Atg4 cysteine protease which is then activated by the Atg7 E1-like enzyme and transferred to the Atg3 E2 enzyme. LC3 is in turn conjugated to the lipid phosphatidylethanolamine and embedded into the membranes for autophagic vesicle expansion [105-107]. Moreover, c-FLIP or vFLIP plays a role in autophagy [81,85,87], demonstrating that these proteins suppress autophagy by preventing Atg3 from binding and processing LC3. Therefore, FLIP expression effectively suppresses cell death with autophagy resulting from treatment with rapamycin, an effective anti-cancer drug. Lee et al. [81] also initially identified an autophagy pathway checkpoint by which c-FLIP and vFLIP limit the Atg3-mediated step of LC3 conjugation and regulate autophagosome biosynthesis. Moreover, these authors identified the FLIP-derived short peptides (a DED1 alpha2-helix ten amino acid [alpha2] peptide or a DED2 alpha4-helix twelve amino acid [alpha4] peptide of FLIP) which individually bind to FLIP and Atg3, effectively inhibit Atg3-FLIP interaction without affecting Atg3-LC3 interaction, and induce significant growth inhibition and cell death with autophagy. Similarly, a recent study also provides evidence that anti-FLIP peptides induce autophagy by inactivating the anti-autophagy activity of c-FLIP, and that this is associated with their antiviral effects against enterovirus 71 (EV71) [87]. These peptides may potentially be useful as anticancer therapies.

## c-FLIP Activates Cytoprotective and Proliferation Pathways

c-FLIP activates several cytoprotective signaling pathways involved in regulating cell survival, proliferation, and carcinogenesis. Overexpression of c-FLIPL activates NF-κB and ERK signaling by binding to adaptor proteins in each pathway, such as TNFR-associated factors 1 (TRAF1) and 2 (TRAF2), Receptor-Interacting Protein 1 (RIP), and Raf-1 [108-110] (Figure 3). The caspase-8 processed N-terminal fragment of c-FLIPL (p43cFLIP) is more efficient than c-FLIPL at recruiting TRAF2 and RIP1, leading to more robust NF-κB activation [110,111]. In non-apoptotic cells, c-FLIP and the procaspase-8 heterodimer and result in a NH2-terminal fragment of c-FLIP (p20-FLIP) which is the key mediator of NF-κB activation by binding to the IKK complex [112]. c-FLIP may also provide the molecular switch from Fas-triggered apoptosis to Fas-promoting cell proliferation [113]. Recently, Golan-Gerst et al. [114] demonstrated that c-FLIP silencing inhibited Fas-induced proliferation of fibrotic lung myofibroblasts and made them susceptible to Fas-induced apoptosis. Piao et al. [115] recently demonstrated that c-FLIP expression controls the homeostasis of Intestinal Epithelial Cells (IEC) and hepatocytes by preventing cell death induced by TNF-α, FasL, and TRAIL. The mammalian target of rapamycin (mTOR) is known to positively regulate cell proliferation and survival through the formation of complexes with raptor (mTOR complex 1; mTORC1) or rictor (mTOR complex 2; mTORC2). Recent results revealed that mTORC2 stabilizes c-FLIPS, hence this cell proliferation/survival signaling complex regulates death receptor-mediated apoptosis [116]. Recently, Jing et al. [117] demonstrated that the H204 residue of c-FLIPL binds to calmodulin (CaM) and a point mutation at this amino acid was found to markedly reduce CaM binding, which mediates the anti-apoptotic function of c-FLIPL in cholangiocarcinoma cells. Further characterizing the CaM/c-FLIP interaction may provide a new therapeutic target for developing anticancer therapies [117]. By co-immunoprecipitation experiments, Quintavalle et al. [118] demonstrated that the interaction between Akt and c-FLIPL enhances the anti-apoptotic functions of Akt by modulating Gsk3-β activity. Moreover, these authors have shown that c-FLIPL overexpression interferes with Gsk3-β phosphorylation levels and induces resistance to TRAIL in cancer cells. DNA-PK/Akt pathway is also reported to play a role in expression of c-FLIP [119]. Moreover, siRNA-mediated suppression of DNA-PK or treatment with 4,5-dimethoxy-2-nitrobenzaldehyde (DMNB), an inhibitor of DNA-PK, led to decreased phosphorylation of Akt and Bad (a target molecule of Akt), increased expression of DR4/DR5, and downregulation of c-FLIP [88]. Furthermore, c-FLIPL directly interacts with a JNK activator, MAP kinase kinase 7 (MKK7), in a TNFα-dependent manner and inhibits the interactions of MKK7 with MAP/ERK kinase kinase 1 (MEKK1), apoptosis signal-regulating kinase 1, (ASK1) and TGF-β-activated kinase 1. This interaction of c-FLIPL with MKK7 might selectively suppress JNK activation [120]. c-FLIP may also regulate several potentially harmful signaling pathways including the production of inflammatory cytokines, tumor cell migration and metastasis, and the activation of transcription factors critical during tumorigenesis [121-123]. c-FLIP overexpression can alter cell cycle progression and enhance cell proliferation and carcinogenesis [117]. Increased expression of c-FLIPL inhibited the ubiquitination and proteasomal degradation of β-catenin, resulting in increased cyclin D1, colony formation, and invasive activity in prostate cancer cells. The c-FLIP/β-catenin/cyclin D1 signals contributing to colony formation and invasion were reversed by selectively silencing c-FLIP expression [93]. Similarly, c-FLIP, in cooperation with FADD, enhances canonical Wnt signaling by inhibiting proteasomal degradation of β-catenin [123]. c-FLIP overexpression is also significantly related to the presence of high-risk human papillomavirus (HR-HPV) infection during the progression of cervical squamous cell cancer, and c-FLIP is an early marker of cervical carcinogenesis [124]. Moreover, HPV16 E2 protein interacts with abrogates the apoptosis inhibitory function of c-FLIP and renders cervical cancer cell lines hypersensitive to Fas/FasL apoptosis [71].

## c-FLIP and Cancer Treatment

The roles of c-FLIP isoforms as proteins that cause resistance to pro-cell death signals, cytokines, and anticancer drugs have been established [33]. Moreover, small interfering RNAs (siRNAs) that specifically silence the expression of c-FLIPL in diverse human cancer cell lines have been shown to enhance TRAIL-induced DISC recruitment and increase the efficacy of chemotherapeutic agents, thereby elevating apoptosis. Furthermore, small molecules causing degradation of c-FLIP as well as decreasing mRNA and protein levels of c-FLIPL and c-FLIPS splice variants have been found, and much effort is focused on developing other c-FLIP-targeted cancer therapies. c-FLIP therapeutic intervention aimed at inhibiting its transcription and posttranscriptional changes is critical for developing anticancer agents [20,34]. Because of significant resemblance to caspase-8 (Figure 2), c-FLIP protein is a very difficult target for drugs that inhibit its function, since small molecules capable of blocking its recruitment to the DISC would also likely inhibit recruitment of caspase-8 to this complex, thereby inhibiting apoptosis. Therefore, to reduce or inhibit c-FLIP expression, small molecules which target c-FLIP without inhibiting caspases-8 and -10 are needed. Diverse classes of agents that decrease c-FLIP expression and sensitize cancer cells to TRAIL or anticancer drugs have been reviewed [19,34,125]. These include some conventional anticancer drugs including cisplatin, doxorubicin, actinomycin D, cycloheximide, camptothecin, 9-NC, and topotecan; histone deacetylase (HDAC) inhibitors; the inhibitors of MEK1/2, PKC and PI3K, and numerous other compounds [19,34,80,125]. These agents affect c-FLIP transcription, trigger c-FLIP degradation through the ubiquitin-proteasome system, or decrease c-FLIP translation. Moreover, DNA damaging agents are promising drugs with regard to downregulating levels of c-FLIP variants [77-126,127]. Downregulating c-FLIP variants by small molecule therapeutics may be a potential strategy for developing agents to treat diseases in which this protein plays a role in preventing apoptosis, necroptosis, and autophagy. However, downregulation of c-FLIP in various cell types has induced significant cell death in healthy cells.

## Conclusions

c-FLIP is involved in inhibiting apoptosis, programmed necroptosis (necrosis) and autophagy. c-FLIP variants induce resistance to death receptor ligands and chemotherapeutic agents in various cancer cells. Moreover, c-FLIP upregulation has been correlated with a poor clinical outcome. Therefore, c-FLIP isoforms may serve as targets for counteracting therapy-resistant human malignancies. Hence, c-FLIP may be a clinically relevant biomarker and a molecular target for developing therapeutics for various diseases. Various classes of agents can downregulate c-FLIP expression. To reduce or inhibit c-FLIP expression, small molecules which target c-FLIP without inhibiting caspases-8 and -10 are needed. Compounds that inhibit or downregulate c-FLIP mRNA expression or cause degradation of c-FLIP at the protein level through proteasome degradation will be of particular interest.
